# The prognostic influence of bcl-2 in malignant glioma

**DOI:** 10.1038/sj.bjc.6600217

**Published:** 2002-06-17

**Authors:** F E McDonald, J W Ironside, A Gregor, B Wyatt, M Stewart, R Rye, J Adams, H W W Potts

**Affiliations:** Northern Centre for Cancer Treatment, Newcastle General Hospital, Westgate Road, Newcastle upon Tyne NE4 6BE, UK; Departments of Pathology and Clinical Oncology, Western General Hospital, Crewe Road, Edinburgh EH4 2XU, UK; ICRF Trials Office, Western General Hospital, Crewe Road, Edinburgh EH4 2XU, UK; ICRF Medical Statistics Group, Institute of Health Sciences, PO Box 777, Headington, Oxford OX3 7LF, UK; Cancer Research UK London Psychosocial Oncology Group, St Thomas' Hospital, London SE1 7EH, UK

**Keywords:** high grade glioma, bcl-2, multivariate analysis

## Abstract

The bcl-2 gene is one of a complex group of genes which control programmed cell death. Bcl-2 acts to extend cell survival by blocking apoptosis, and thereby may influence tumour prognosis. This study of 187 high grade gliomas reviews clinicopathological prognostic features and the relationship to bcl-2 expression. Bcl-2 immunostaining was assessed in 159 specimens from these patients, by scoring systems of 0 to 3 for intensity of scoring and proportion of cells staining. Age, histology, pre- and post-operative performance status were found to be strongly predictive of survival (log rank test *P*<0.0001). The type of surgery performed did not influence survival in this group of patients. The expression of bcl-2 had a significant relationship with survival (univariate Cox model *P*=0.0302, hazard ratio 0.8, 95% confidence interval 0.65–0.98), with increased staining associated with improved survival. Multivariate analysis showed performance status, histology and proportion of cells staining for bcl-2 to be independently predictive of survival. Bcl-2 staining was not related to histological grade of tumours.

*British Journal of Cancer* (2002) **86**, 1899–1904. doi:10.1038/sj.bjc.6600217 www.bjcancer.com

© 2002 Cancer Research UK

## 

Gliomas form the most common group of primary brain tumours, accounting for 40% of all CNS neoplasms. Malignant gliomas (glioblastoma multiforme and anaplastic astrocytomas) are most common and carry the worst prognosis. However, malignant gliomas are a heterogeneous group of neoplasms, with a varied survival. Accurate identification of the factors influencing prognosis in malignant gliomas is important in selecting appropriate treatment and in evaluating its effectiveness. Moreover, the investigation of factors associated with particular prognosis may elucidate the underlying biological mechanisms involved in the development or progression of the tumour. Bcl-2 is one of a family of interacting proteins involved in the regulation of controlled cell death, apoptosis (Korsmeyer, 1992; McDonnell *et al*, 1996). Bcl-2 appears to protect against apoptosis by preventing cells from responding to stimuli to initiate apoptosis. The bcl-2 protein forms homo- and hetero-dimers with another protein in this family, Bax. The ratio of activity of the pro-apoptotic Bax to the anti-apoptotic bcl-2 determines the response to apoptotic stimuli; a decrease in the ratio of bcl-2 to Bax protein renders cells more vulnerable to apoptosis (Oltvai *et al*, 1993). P53 acts to cause this effect (Miyashita *et al*, 1994).

Radiation and chemotherapy exert their effects largely through the induction of apoptosis, so excessive bcl-2 activity may render them less effective. Apoptosis is a recognised feature of malignant glioma (Ellison *et al*, 1995), but the relationship of bcl-2 expression to this is unclear. Increasing numbers of apoptotic bodies have been shown to correlate with increasing grade of malignancy in gliomas (Ellison *et al*, 1995). However, whether bcl-2, as a protector from apoptosis, shows an inverse relationship to this is indeterminate, with conflicting results (Nakasu *et al*, 1994; Krishna *et al*, 1995; Weller *et al*, 1995). It is also unclear whether bcl-2 expression relates to grade of CNS tumours, though there are suggestions that it may. Nakasu *et al* (1994) and Ehrmann (1997) both found lower grade tumours to have higher bcl-2 expression, but other groups have failed to confirm these results (Schiffer *et al*, 1996; Newcomb *et al*, 1997, 1998).

In malignant glioma inherent prognostic features appear to exert a much stronger influence on survival than treatment effects. Age in particular seems to have an overriding effect on survival, independent of other factors (Burger and Green, 1987; MRC Working Party, 1990). Duration of symptoms, performance status, presence and duration of fits as a first symptom, and degree of neurological impairment at presentation have all been recognised as factors which influence prognosis (Cohaden *et al*, 1985; MRC Working Party, 1990; Hutton *et al*, 1992). In addition, the prognostic significance of the extent of surgical resection has been identified in several series (MRC Working Party, 1990; Devaux *et al*, 1993), and histological grade and features, in particular necrosis, appear to be major prognostic determinants (Cohaden *et al*, 1985; Seung Kim *et al*, 1991; Salmon *et al*, 1994).

This study therefore aimed to evaluate the prognostic effect of bcl-2 in the context of a patient population where these inherent factors are well characterised.

## MATERIALS AND METHODS

Edinburgh provides a tertiary referral centre for neuro oncology with a multidisciplinary unit which was established in 1987. Three hundred and fifty-six patients were seen in the first 6 years, and none have been lost to follow-up. This group represents approximately 60% of the incident cases diagnosed in the geographical area covered by the unit. A database of all patients referred to the neuro oncology unit was established, collecting information on demographics, clinical presentation, Karnofsky performance status (KPS), pathological diagnosis, treatment and subsequent follow up. The management policy for malignant gliomas in the unit is for maximal surgical resection compatible with safety, followed by radiotherapy to the initial tumour site with generous margins. For high grade tumours the dose used is 60 Gy in 30 daily fractions over 6 weeks. Chemotherapy is not used routinely in the primary or adjuvant setting, but is used for selected patients at relapse, usually as part of a clinical study.

Surgical biopsy specimens from the 187 high grade glioma patients diagnosed since 1987 were retrieved from the files of the Neuropathology Laboratory, Edinburgh. Of these, there was sufficient material in 159 cases to allow additional investigations to be performed and these cases were then reviewed using the WHO Classification (Kleihues *et al*, 1993) in relation to the earlier diagnosis and entered into the study (79 glioblastoma and 80 anaplastic astrocytomas). From the surgical blocks, sections were stained by haemotoxylin and eosin for review and most of the cases had the diagnosis confirmed using immunocytochemistry for glial acidic protein (DAKO, UK) in a standard immunoperoxidase technique. Sections were chosen to be representative of tumour rather than containing large numbers of reactive cells. Immunostaining for bcl-2 was performed by a standard ABC method using a mouse anti-human bcl-2 primary antibody. A normal tonsil was used as a positive control, and a negative control with omission of the primary antibody.

**Figure 1:**
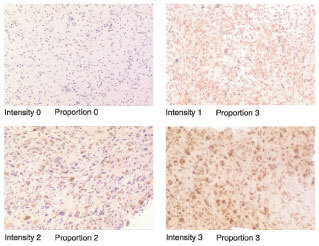
Examples of Bcl-2 immunostaining scoring systems.

Immunostaining was assessed by semiquantitative scoring systems for percentage of cells staining and intensity of staining. Both were scored on scales of 0 to 3. For percentage of cells staining, a score of 0 represented no cells staining, a score of 1 represented less than 25%, a score of 2, 25 to 50%, and a score of 3 over 50% of cells staining. To establish a scale for intensity of staining an initial pilot group of sections was examined to observe the range of staining. A semiquantative scale of 0 to 3 was then used to assess the intensity of staining. See Figure 1

for example of staining scores.

Gliomas are often heterogeneous in their composition, and where bcl-2 staining was variable an attempt was made to give an overall representative score. The sections were scored by two observers (FE McDonald and JW Ironside), blind to other data.

### Statistical methods

Descriptive analysis was firstly performed on the clinical data. Kaplan–Meier plots of survival were produced, and the logrank test performed. As bcl-2 staining intensity and proportion are both ordered values, trend analysis using univariate Cox models was performed. Multivariate analysis by backward stepwise Cox regression analysis was then performed to explore the combined effect of these variables on survival (Cox and Oakes, 1984)

## RESULTS

### Patient characteristics

This study examined 187 high grade gliomas. The mean age was 51 years (s.d. of 13.6), range 8 to 75 years. The mean age of patients with glioblastoma multiforme was 54.5 years, and of anaplastic astrocytoma 48 years. Sixty-one per cent of ‘high grade’ patients were male and 31% female.

Sixty-eight patients had a biopsy of the tumour, 31 had a macroscopically complete resection, 86 had a partial resection. All except six had post-operative radiotherapy (Table 1

**Table 1:**
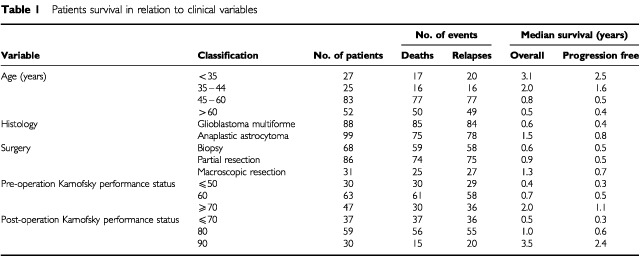
Patients survival in relation to clinical variables

).

The mean age of patients with Karnofsky performance status (KPS) 90 was 38.6 years (s.d. 10.6), KPS 80 was 53.7 years (s.d. 11.7) and KPS 70 or less was 57.1 years (s.d. 9.9), suggesting age and performance status are closely linked. Patients with glioblastoma multiforme had a slightly worse pre and post operative performance status (mean KPS 58.76 and 76.53 respectively) compared to anaplastic astrocytoma patients (mean KPS 65.59 and 81.48 respectively).

### Bcl-2 analysis

Bcl-2 staining intensity did not appear to have any significant relationship to the other clinicopathological variables; in particular it did not seem to be related to tumour grade (Fisher's Exact *P*=0.443). Proportion of cells staining also did not show any significant relationship to the other variables, including histology (Fisher's Exact *P*=0.612).

### Survival analysis

**Figure 2:**
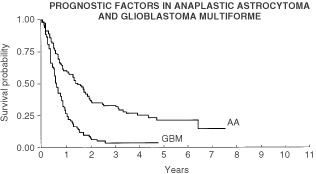
Influence of histology on overall survival.

**Figure 3:**
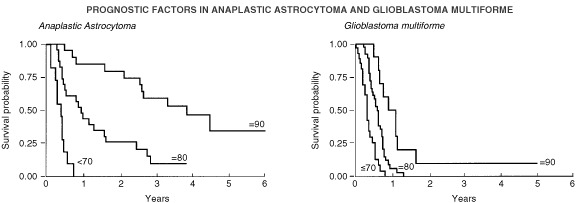
Influences of histology and post-operative Karnofsky performance status on progression free survival.

Survival was analysed with respect to all the collected variables. The median overall survival for the group was 10.3 months. The median progression free survival was 6.4 months. For glioblastoma multiforme patients the mean progression free survival was 6.8 months and overall survival was 10.1 months. For anaplastic astrocytomas the mean progression free survival was 19 months and overall survival was 24.7 months (Figure 2

). The influence of post operative performance status on survival is marked. The mean progression free survival for patients with KPS 90 was 29.8 months, for patients with KPS 80 was 10.4 months, and for patients with a KPS 70 or less 4.2 months (Figure 3

).

**Figure 4:**
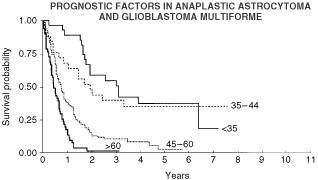
Influence of age on overall survival.

The log rank test identified age as a strongly statistically significant predictor of survival (Figure 4

), with *P*<0.0001 for both overall and progression-free survival. Histology and both pre- and post-operative performance status were also strongly predictive for survival with *P* values of <0.0001. In contrast the type of surgery was not a statistically significant predictor of survival, with *P* values of 0.36 for overall survival and 0.61 for progression-free survival.

**Figure 5:**
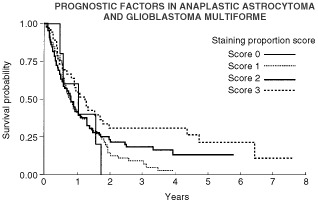
Influence of proportions of cells staining for bcl-2 on overall survival.

Univariate Cox models showed a statistically significant relationship between the proportion of cells staining for bcl-2 and overall survival, with a *P* value of 0.0302. This model gave a hazard ratio of 0.8 (95% confidence interval 0.65–0.98), so a higher proportion staining is associated with a better prognosis (Figure 5

). There did not seem to be a relationship to progression free survival however (*P*>0.1). The models did not show any significant relationship between the intensity of staining and either overall or progression free survival (*P* values >0.1).

### Multivariate analysis

Cox's regression analysis was performed on all five clinical variables with respect to overall survival. This identified histology and post-operative performance status as independent important predictors of survival. Histology of glioblastoma multiforme as opposed to anaplastic astrocytoma gave a hazard ratio of 0.41 with 95% confidence interval of 0.26 to 0.65 and *P* value <0.001. Post-operative performance status ⩽70 *vs* 80 gave a hazard ratio of 0.34 with 95% confidence interval 0.22 to 0.54 and *P* value <0.001 for overall survival. Post-operative performance status ⩽70 *vs* 90 gave a hazard ratio 0.08 with 95% confidence interval 0.04 to 0.15 and *P* value <0.001.

For progression-free survival the same two variables were identified. Histology gave a hazard ratio of 0.41 with 95% confidence interval 0.26 to 0.64 and *P* value <0.001. Post-operative performance status ⩽70 *vs* 80 gave a hazard ratio of 0.29 with 95% confidence interval 0.18 to 0.46 and *P* value <0.001. Post-operative performance status ⩽70 *vs* 90 gave a hazard ratio of 0.08 with 95% confidence interval 0.04 to 0.16 and *P* value <0.001. Post-operative performance status and age were found to be highly correlated, so it is likely that the effect of age is hidden by the influence of performance status.

Bcl-2 staining was then included in the Cox modelling (Table 2

**Table 2:**
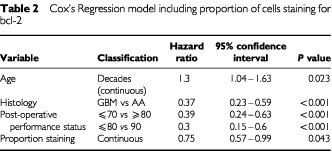
Cox's Regression model including proportion of cells staining for bcl-2

). The resulting model produced similar conclusions in relation to the clinical variables, but in addition identified the proportion of cells staining for bcl-2 as a statistically significant independent predictor of overall but not progression free survival. The hazard ratio for bcl-2 proportion treated as a continuous variable was 0.75 with 95% confidence interval 0.57–0.99, and *P* value 0.043. Intensity of bcl-2 staining did not appear to be predictive of survival.

## DISCUSSION

In common with other groups, multivariate analysis of the clinical and histological data identified tumour grade and performance status as independently significant prognostic factors. In addition, in univariate analysis, age was identified as a strongly significant predictor of survival, but the close correlation with performance status seems to have masked this effect in multivariate analysis. This study confirms the predictive value of the WHO grading system. The type of surgery performed did not predict survival in this group of patients, however.

Analysis of bcl-2 staining showed that the proportion of cells staining was independently predictive of survival, but that the intensity was of no prognostic value. This intensity of staining result may reflect several factors. It is possible that the variability in intensity of staining is an artefact of staining technique, which may depend on variables in tissue preservation, fixation etc., although all specimens were processed in a standard way in the Neuropathology Laboratory.

The proportion of cells staining correlated with the overall (though not progression free) survival, with greater staining predicting longer survival. It was particularly interesting that this was independent of histology.

A common limitation of studies on gliomas is that biopsies may be small and unrepresentative because of tumour heterogeneity. In addition, there may be a further sampling error when blocks are chosen for staining. In this study it was noted that seven of the sections were particularly small and may not be representative. A further possible criticism is that no attempt was made to distinguish the type of cells, neoplastic or reactive, which were positively staining.

Several previous studies have also examined bcl-2 staining in gliomas. Newcomb *et al* (1998) examined several growth control genes including bcl-2 in 80 patients with glioblastoma multiforme. They used a similar four step score to assess the frequency of immunopositive cells, but only analysed by positive verses negative staining. They found no difference in survival according to bcl-2 staining. Ehrmann *et al* (1997) looked at bcl-2 immunohistochemical expression in 42 patients with astrocytomas, of which 23 were malignant gliomas. Again a scoring system was used, but analysis was based on positive *vs* negative staining. In this small group no relationship to overall survival was found, although higher bcl-2 staining was noted in lower grade tumours. Schiffer *et al* (1996) examined bcl-2 expression in 100 CNS tumours, which included 21 grade 3 or four tumours, and found no correlation with survival in each tumour type.

Newcomb *et al* (1997) also assessed bcl-2 expression, relating it to survival and p53 expression in 58 astrocytomas from 21 paediatric patients and 37 adult patients. They found no relationship between bcl-2 staining and survival in the paediatric patients, but found the bcl-2 negative WHO grade IV tumours in adults had a shorter median survival. Our study examined a larger group of malignant gliomas, giving more statistical power to detect survival effects within this group than these other studies.

Strik *et al* (1999) looked at bcl-2 expression in 37 cases of glioblastoma multiforme at first resection and then again at recurrence. They found bcl-2 positivity in a high percentage of initial (91%) and recurrent tumours (97%). They suggest that a shift in the bcl-2 rheostat may lead to resistance to apoptosis, but found this in both patients treated with radiotherapy with or without chemotherapy, and in patients who had received no treatment, in favour of natural resistance to apoptosis rather than acquired resistance due to the selective pressures of therapy. They also found that patients with initial negative or weak bcl-2 expression had a trend to better prognosis than those with an intermediate or strong bcl-2 expression (12 *vs* 8 months median progression free survival), though this result was not statistically significant in their small group of patients.

The finding that increased bcl-2 staining confers a survival advantage is contrary to much of our understanding of bcl-2. Bcl-2 prevents apoptosis, and may thereby cause neoplastic cell population expansion. Increased bcl-2 expression would therefore be expected to confer a worse prognosis. In addition, radiotherapy and chemotherapy induce apoptotic cell death, and an excess of bcl-2 should render them less effective. Bcl-2 does not prevent chemotherapy induced cell cycle arrest, but prolongs survival during this period, so that on withdrawal of the drug proliferation can resume. Thus it changes the drug from being cytotoxic to cytostatic. Chemotherapeutic agents also damage DNA and bcl-2 prevents this from leading to cell death, allowing mutations to persist.

It has long been recognised that glioblastomas have frequent apoptotic bodies. Ellison *et al* (1995) correlated apoptotic index with increasing grade of malignancy, and rate of proliferation assessed by Ki-67 labelling. They found higher bcl-2 staining in lower grade tumours. There was a trend for apoptotic index to be lower in bcl-2 positive than negative tumours, but this was non significant. They also examined the interaction of bcl-2 with the promoter of apoptosis, p53. From their results they hypothesised that upregulation of bcl-2 is an advantage in tumours where increased wild type p53 promotes apoptosis in response to DNA damage, but where p53 is disabled, this upregulation of bcl-2 is not required to increase tumour growth. Alderson *et al* (1995) produced some evidence to support this theory, showing bcl-2 expression was more common in gliomas with wild type p53. Newcomb *et al* (1997) failed to confirm this in their series of gliomas, however.

In other tumours bcl-2 positivity has not been clearly correlated with tumour aggressiveness. In prostate cancer it appears to be a late event, occurring with androgen independence (McDonnell *et al*, 1992; Colombel *et al*, 1993), whereas in breast and colonic cancer it appears to be an early event (Hague *et al*, 1994; Joensuu *et al*, 1994; Leek *et al*, 1994). In neuroblastoma bcl-2 expression seems to correlate with the morphology and differentiation of neuroblastoma cells *in vitro*, greater bcl-2 expression being associated with more neuroblastic cells (Reed *et al*, 1991).

Studies in, for example, myeloid leukaemia, carcinoma of prostate and large cell Non Hodgkins lymphoma, have found bcl-2 expression to be associated with a poor response to treatment, and a shorter disease free and overall survival (Colombel, 1993; Reed *et al*, 1996). However, Silvestrini *et al* (1994), found bcl-2 expression to correlate with a good prognosis in breast cancer. Reed *et al* (1996) discussed this paradoxical result, hypothesising that it is a reflection of the complex interaction between the family of genes which control apoptosis, and in particular the ratio of activity of the proapoptotic gene Bax to the anti apoptotic bcl-2. A decrease in the ratio of bcl-2 to Bax protein renders cells more vulnerable to apoptosis (Borner, 1996). P53 acts to cause this effect and other members of the bcl-2 family also act in concert. Reed *et al* (1996) suggest that in Silvestrini's group of patients reductions in bcl-2 were tolerated because of diminished Bax protein, keeping the ratio in favour of apoptosis, and conferring a good prognosis.

Krawejski *et al* (1995) showed that loss of BAX expression was strongly associated with loss of bcl-2 immunopositivity in a small group of metastatic breast cancer patients. Reduced Bax (and bcl-2) immunostaining was associated with poor responses to chemotherapy and shorter overall survival. Similarly, in cervical cancer, Crawford *et al* (1998) found that bcl-2 was associated with a better 5 year survival. They found p53 positivity was associated with a survival disadvantage but Bax was not predictive of survival in their group of patients.

In our study bcl-2 staining was associated with better survival. We would hypothesise that this reflects interaction between bcl-2 and the other factors controlling apoptosis. It would therefore be of interest to examine Bax and p53 expression in this group of patients to further clarify these interactions. If therapeutic manipulations of the apoptotic pathway are to be effective then the complexity of the pathway and the interacting members must be considered.

In conclusion, this study reviewed the prognostic factors in high grade glioma and found, in common with previous studies, that age, performance status and histology are predictive of survival. We have identified the proportion of cells staining for bcl-2 as a statistically significant prognostic factor in high grade glioma, independent of histological grade. This finding warrants further investigation, both to confirm this result in another group of patients and to elucidate the underlying mechanisms influencing glioma cell apoptosis.

## References

[bib1] AldersonLMCastlebergRLHarshGRLouisDNHensonJW1995 Human gliomas with wild type p53 express bcl-2 Cancer Res 55 9991001 7867012

[bib2] BornerC1996 Diminished cell proliferation associated with the death-protective activity of bcl-2 J Biol Chem 271221269512698 8663032 10.1074/jbc.271.22.12695

[bib3] BurgerBCGreenS1987 Patient age, histological features, and length of survival in patients with glioblastoma multiforme Cancer 59 16171625 3030531 10.1002/1097-0142(19870501)59:9<1617::aid-cncr2820590916>3.0.co;2-x

[bib4] CohadenFAouadNRougierAVitalCRivelCDartiguesJF1985 Histologic and non-histologic factors correlated with survival time in supratentorial astrocytic tumours J Neuro-Oncology 3 105111 10.1007/BF02228885 4031969

[bib5] ColombelMSymmansFGilSO'TooleKMChopinDBensonMOlssonCAKorsmeyerSButtyanR1993 Detection of the apoptosis suppressing oncogene bcl-2 in hormone refractory human prostate cancer Am J Pathol 143 390400 7688182 PMC1887010

[bib6] CoxDROakesD1984 Analysis of survival dataLondon: Chapman and Hall

[bib7] CrawfordRAFCaldwellCIlesRKLoweDShepherdJHChardT1998 Prognostic significance of the bcl-2 apoptotic family of proteins in primary and recurrent cervical cancer Br J Cancer 78 210214 9683295 10.1038/bjc.1998.466 PMC2062899

[bib8] DevauxBCO'FallonJRKellyPJ1993 Resection, biopsy, and survival in malignant glial neoplasms J Neurosurg 78 767775 8468607 10.3171/jns.1993.78.5.0767

[bib9] EhrmannJrJKolarZVojtesekBKalaMKomendaSOultonA1997 Prognostic Factors in astrocytomas: Relationship of p53, MDM-2, Bcl-2 and PCNA immunohistochemical expression to tumor grade and overall patient survival Neoplasia 44 299304 9473789

[bib10] EllisonDWSeartPVGatterKCWellerRO1995 Apoptosis in cerebral astrocytic tumours and its relationship to expression of the bcl-2 and p53 proteins Neuropath Applied Neurobiol 21 352361 10.1111/j.1365-2990.1995.tb01070.x 7494604

[bib11] HagueAMoorghenMHicksDChapmanMParaskevaC1994 Bcl-2 expression in human colorectal adenomas and carcinomas Oncogene 9 33673370 7936663

[bib12] HuttonJLSmithDFSandemannDFoyPMShawMDMWilliamsIRChadwickDW1992 Development of prognostic index for primary supratentorial intracerebral tumours J Neurol, Neurosurg Psychiat 55 271274 1583511 10.1136/jnnp.55.4.271 PMC489038

[bib13] JoensuuHPylkkanenLToikkanenS1994 Bcl-2 protein expression and long term survival in breast cancer Am J Pathol 145 11911198 7977649 PMC1887415

[bib14] KleihuesPBurgerPCSheithauerBW1993 Histological Typing of Tumours of the Central Nervous System: World Health Organisation International Histological Classification of TumoursBerlin: Springer

[bib15] KorsmeyerS1992 Bcl-2 initiates a new category of oncogenes: regulators of cell death Blood 80 879886 1498330

[bib16] KrajewskiSBlomvqvistCFranssilaKKrajewskaMWaseniusV-MNiskanenEReedJC1995 Reduced expression of the pro-apoptotic gene Bax is associated with poor response rates to combination chemotherapy and shorter survival in women with metastatic breast adenocarcinoma Cancer Res 55 44714478 7671262

[bib17] KrishnaMSmithTWRechtLD1995 Expression of bcl-2 in reactive and neoplastic astrocytes: lack of correlation with presence or degree of malignancy J Neurosurg 83 10171022 7490615 10.3171/jns.1995.83.6.1017

[bib18] LeekRDKaklamanisLPezzellaFGatterKCHarrisAL1994 Bcl-2 in normal human breast and carcinoma, association with oestrogen receptor positive, epidermal growth factor negative tumours and in situ cancer Br J Cancer 69 135139 8286195 10.1038/bjc.1994.22 PMC1968762

[bib19] McDonnellTJTroncosoPBrisbaySMLogothetisCChungLWKHseihJTTuSMCampbellML1992 Expression of the proto-oncogene bcl-2 in the prostate and its association with the emergence of androgen-independent prostate cancer Cancer Res 52 69406944 1458483

[bib20] McDonnellTJBehamASarkissMAndersonMMLoP1996 Importance of the Bcl-2 family in cell death regulation Experentia 52 10081017 10.1007/BF01920110 8917732

[bib21] MiyashitaTKrajewskiSKrajewskiMWangHG1994 Tumor suppressor p53 is a regulator of bcl-2 and bax in gene expression in vitro and *in vivo* Oncogene 9 17991805 8183579

[bib22] MRC: A report of the Medical Research Council Brain Tumour Working Party1990 Prognostic factors for high grade malignant glioma: Development of a prognostic index J Neuro-Oncol 9 4755 10.1007/BF00167068 2213115

[bib23] NakasuSNakasuYNiokaHNakajimaMHandaJ1994 Bcl-2 protein expression in tumours of the central nervous system Acta Neuropathol 88 520526 7879598 10.1007/BF00296488

[bib24] NewcombEBhallaSKParrishCLHayesRLCohenHMillerDC1997 Bcl-2 protein expression in astrocytomas in relation to patient survival and p53 gene status Acta Neuropathol 94 369375 9341939 10.1007/s004010050721

[bib25] NewcombEWCohenHLeeSRBhallaSKBloomJHayesRLMillerDC1998 Survival of patients with glioblastoma multiforme is not influenced by altered expression of P16, p53, EGFR, MDM2 or Bcl-2 genes Brain Pathol 8 655667 9804374 10.1111/j.1750-3639.1998.tb00191.x PMC8098514

[bib26] OltvaiZMillimanCKersmeyerSJ1993 Bcl-2 heterodimerises in vivo with a conserved homolog, Bax, that accelerates programmed cell death Cell 74 609619 8358790 10.1016/0092-8674(93)90509-o

[bib27] ReedJCMeisterLTanakaSCuddyMYumSGeyerCPleasureD1991 Differential expression of bcl-2 protooncogene in neuroblastoma and other human tumour cell lines of neural origin Cancer Res 51 65296538 1742726

[bib28] ReedJCMiyashitaTTakayamaSHong-GongWSatoTKrawjewskiSAime-SempeCBodrugSKitadaSHanadaM1996 Bcl-2 family proteins: Regulators of cell death in the pathogenesis of cancer and resistance to therapy. (Review) J Cell Biochem 60 2332 8825412 10.1002/(SICI)1097-4644(19960101)60:1%3C23::AID-JCB5%3E3.0.CO;2-5

[bib29] SalmonIDewitteOPasteelsJ-LFlament-DurandJBrotchiJVereerstraetenPKissR1994 Prognostic scoring in adult astrocytic tumors using patient age, histopathological grade, and DNA histogram type J Neurosurg 80 877883 8169628 10.3171/jns.1994.80.5.0877

[bib30] SchifferDCavallaPMigheliAGiordanaMTChiado-PiatL1996 Bcl-2 distribution in neuroepithelial tumors: an immunohistochemical study J Neuro-oncol 27 101109 10.1007/BF00177472 8699231

[bib31] Seung KimTHallidayALHedley-WhiteTConveryK1991 Correlates of survival and the Daumas-Duport grading system for astrocytomas J Neurosurg 74 2737 1984503 10.3171/jns.1991.74.1.0027

[bib32] SilvestriniRVeneroniSDaidonemMBeniniEBoracchiPMezzettiMDiFronzoG1994 The bcl-2 protein: a prognostic indicator strongly related to p53 in lymph node negative breast cancer patients J Natl Cancer Inst 86 499504 8133533 10.1093/jnci/86.7.499

[bib33] StrikHDeiningerMStrefferJGroteEWickboldtJDichgansJWellerMMeyermannR1999 Bcl-2 Family protein expression in initial and recurrent glioblastomas: modulation by chemoradiotherapy J Neurol Neurosurg Psych 67 763768 10.1136/jnnp.67.6.763 PMC1736652 10567494

[bib34] WellerMMalipeiroUAgussiAReedJCFontanaA1995 Protooncogene bcl-2 gene transfer abrogates Fas/APO-1 Antibody-mediated apoptosis of human malignant glioma cells and confers resistance to chemotherapeutic drugs and therapeutic irradiation J Clin Invest 95 26332643 7539458 10.1172/JCI117965 PMC295946

